# Long-term prognostic impact of cystatin c on acute coronary syndrome octogenarians with diabetes mellitus

**DOI:** 10.1186/1475-2840-12-157

**Published:** 2013-11-01

**Authors:** Zhenhong Fu, Hao Xue, Jun Guo, Lian Chen, Wei Dong, Luyue Gai, Hongbin Liu, Zhijun Sun, Yundai Chen

**Affiliations:** 1 Department of Cardiology, Chinese People’s Liberation Army General Hospital, 28 Fuxing Road, Beijing, Haidian District 100853, People’s Republic of China

**Keywords:** Acute coronary syndrome, Cystatin C, Octogenarian, Diabetes mellitus, Prognosis

## Abstract

**Objective:**

Cystatin C (Cys C) is a marker of renal dysfunction. Prior studies have shown that blood Cys C is related to the prognosis of coronary heart disease. The aim of the present study was to evaluate the long-term prognostic impact of Cys C on acute coronary syndrome (ACS) octogenarians with diabetes mellitus (DM).

**Methods:**

We enrolled 660 consecutive ACS octogenarians who underwent coronary angiography and were classified into two groups based on diabetes. The baseline characters and Cys C level were measured on admission. Survival curve was calculated using the Kaplan-Meier method. Multivariate Cox regression was used to identify predictors of mortality and of major adverse cardiac events (MACE) rate.

**Results:**

There were 223 and 398 patients in groups DM and non-DM who fulfilled the follow-up. The average follow-up period was 28 (IQR 16–38) months. Diastolic blood pressure (DBP) was lower, ratios of hypertension and chronic renal failure (CRF), fasting blood glucose, HbA1c and Cys C levels were higher in DM group than those in non-DM group (P<0.01). The cumulative survival of DM group was significantly lower than that of non-DM group in the long term (P = 0.018). All cause mortality and MACE of DM group were higher than those of non-DM group (P<0.05). The plasma Cys C concentration (OR = 3.32, 95% CI = 1.18-10.92, P = 0.023) was the uniqueness independent predictor for long-term all cause mortality. The plasma Cys C concentration (OR = 2.47, 95% CI = 1.07-7.86, P = 0.029) and Genesis score (OR = 1.01, 95% CI = 1.00-1.03, P = 0.043) were independent predictors for MACE in DM group. ROC curve analysis showed that the predictive cut-off value of Cys C for mortality of DM group was 1.605 (0.718, 0.704).

**Conclusions:**

Cys C is an independent predictor for long-term mortality and MACE of ACS octogenarians with DM.

## Introduction

The older population represents a growing proportion of the general population. Octogenarian (age *>*80 years old) will increase from the current ratio of 1 in 35 to more than 1 in 12 by the year 2050 [1]. The increasing prevalence of coronary artery disease (CAD) is associated with aging. Compared with younger patients, elderly patients who undergo coronary angiography (CAG) present more complex lesions, higher comorbidity, poorer clinical outcomes and higher mortality [2,3].

Cystatin C (Cys C) is a cysteine protease inhibitor with a low-molecular weight (13 kD) that is produced by all nucleated cells at a constant rate. It is freely filtered across the glomerular membrane and is not influenced by age, sex, muscle mass, exercise or diet [4]. Therefore, the serum Cys C level is a superior marker for the evaluation of renal function compared to other markers such as serum creatinine or creatinine clearance [5]. Over the last few years it has been suggested that high Cys C concentration is related directly to both inflammation and atherosclerosis [6]. Cys C level is also associated with the prognosis of CAD in the general population [7-11]. Diabetes mellitus (DM) is an established risk factor and equivalent of CAD. Patients with CAD and DM are at very high risk, and the prognosis of these patients is poor. Prior studies show a positive relationship of serum Cys C level with the incidence of type 2 DM [12]. The findings strongly suggest Cys C is associated with not only the development of cardiovascular disease (CVD) but also the incidence of type 2 DM. Therefore, we presume that Cys C plays an important role during the short and long term prognosis of an ACS patient with DM.

To date, the prognostic value of Cys C in very old (age *>*80) ACS patients, especially with DM, remains unclear. The prior studies were simply focused on the ACS patients at the age of about 60 years old. Therefore, the aim of the present study is to evaluate long-term prognostic impact of plasma Cys C on Chinese ACS octogenarians with DM.

## Methods

### Study population

From January 2006 to December 2011, a total of 660 consecutive patients with ACS (age > 80 years old), who were referred to our hospital for percutaneous coronary intervention (PCI), were enrolled in this study. The inclusion criteria were: (1) patients with a complete clinical history; (2) a diagnosis of ACS that was classified as unstable angina pectoris (UAP), non-ST-segment elevated myocardial infarction (NSTEMI) or ST-segment elevated myocardial infarction (STEMI) [13] and (3) recent coronary angiography (CAG). Those with acute infection, chronic hepatic dysfunction, nutritional derangements, malignancy, severe valvular heart disease, severe heart failure, dysthyroidism or other severe medical illnesses were excluded.

All patients consented in written to their participation in the study, and the study agreement was approved by the Chinese People’s Liberation Army General Hospital research ethics committee and complied with the Declaration of Helsinki.

### Data collection

The clinical characteristics of all patients were recorded on admission. These included age, gender, heart rate (HR), body mass index (BMI), systolic and diastolic blood pressure (SBP, DBP), eject fraction (EF), diabetes, primary hypertension, hyperlipidemia, previous myocardial infarction (MI), previous stroke, chronic renal failure (CRF), smoking history, and cardiovascular medication. Fasting blood samples were drawn prior to angiography to evaluate blood biochemistry. The fasting blood glucose (FBG), HbA1c, triglyceride (TG), total cholesterol (TC), low density lipoprotein-C (LDL-C), serum creatinine (CRE), urea nitrogen (BUN), and Cys C were analyzed. For all patients, renal function was assessed using the baseline estimated glomerular filtration rate (eGFR). Impaired renal function was defined as an eGFR <60 mL/min/1.73 m^2^. The creatinine was standardized using a calibration equation called Jaffe’s kinetic method [14]:

$$\mathrm{Scr}\left(\mathrm{mg}/\mathrm{dl}\right)=0.795\times \left[\mathrm{enzymatic}\;\mathrm{method}\;\mathrm{Scr},\left(\mathrm{mg}/\mathrm{dL}\right)\right]+0.29$$

The estimated glomerular filtration rate (eGFR) was calculated using the Chinese modified Modification of Diet in Renal Disease (C-MDRD) equation [15]:

$$\begin{array}{l} \mathrm{eGFR}\left(\mathrm{ml}/\min/1.73m^{2}\right)=175\;\times \;\mathrm{standardized}\;\mathrm{creatine}\left(\mathrm{mg}/\mathrm{dl}\right)\\ \;-1.234\;\times \;\mathrm{age}\left(\mathrm{year}\right)-0.179\\ \;\times \;0.79\;\left(\mathrm{if}\;\mathrm{female}\right) \end{array}$$

### Coronary angiography

CAG was performed on all patients after admission. CAD was defined as an obstructive lesion causing ≥ 50% reduction of lumen diameter in at least one of the coronary arteries. The severity of CAD was evaluated using the Gensini score [16].

The 8 variables used in the GRACE risk model included older age, Killip class, systolic blood pressure, ST-segment deviation, cardiac arrest during presentation, serum creatinine level, positive initial cardiac biomarkers, and heart rate. The sum of scores was applied to determine the corresponding all cause mortality in-hospital and from hospital discharge to 6 months [17].

Gensini and GRACE scores for each enrolled patient were recorded by observers who were blinded to the results of laboratory tests and grouping.

### Population grouping and follow-up

Our patients were divided into DM group and non-DM group. The definition of DM was such that a person had an FBG higher than 7 mmol/L, or a 2 hour PBG or random glucose higher than 11.1 mmol/L. All patients were regularly followed at 6-month intervals for the first 12 months, and thereafter every 12 months. All patients were advised to contact the outpatient clinic as soon as possible after a symptomatic event. Long-term outcomes during the follow-up were compared between the two groups.

A major adverse cardiac event (MACE) included any of the following: death for any cause, MI, and need for repeat revascularization (repeat PCI or coronary artery bypass grafting (CABG)).

### Statistical analysis

Continuous variables were expressed as the mean ± standard deviation (SD) or median (with inter quartile range (IQR)). The *t* test was used if continuous variables were normally distributed, while the Wilcoxon two-sample test was used if continuous variables were not normally distributed. Categorical data were summarized as frequency. The chi-square test was used to compare categorical variables. Survival rates were calculated according to the Kaplan-Meier method. Survival time was defined as from the date of coronary angiography to the date of death as verified during the follow-up. Differences between pairs of survival curves were tested by the log-rank test. A Cox proportional hazards model was used to identify predictors of mortality. Odds ratios (ORs) were reported with corresponding 95% confidence intervals (CIs). Receiver operating characteristic (ROC) curves were constructed for discrimination between survived and dead patients. The areas under the curve (AUC) were compared by using Hanley and McNeil method. All P values were two-sided, and a P value < 0.05 was considered statistically significant. Statistical analysis was performed using the Statistical Package for Social Sciences, version 17.0 (SPSS, Chicago, Illinois).

## Results

### Baseline characteristics of patients

Baseline clinical characteristics and clinical event data were fully documented for 621 (621/660, 94.09%) enrolled patients during the follow-up period. 14 patients in DM group and 25 patients in non-DM group were lost during the follow-up because of the wrong telephone number. Of the 621 patients, 223 (35.91%) patients had diabetes and 398 (64.09%) patients did not have diabetes (1: 1.78). The ratio of ACS octogenarians with DM was over one third.

The baseline clinical characteristics of the two groups were shown in Table 1. As compared with those in the non-DM group, patients in DM group had lower DBP (P<0.01) and higher level of FBG (P<0.01) and HbA1c (P<0.01), while other characteristics including age, gender, HR, BMI, TG, TC, LDL-C, SBP, EF, Genesis score and GRACE risk score were not different (P>0.05). The morbidities of hypertension and CRF were higher in DM group than in non-DM group (P<0.01). Among renal function indicators, only plasma Cys C concentration in DM group was higher than that in non-DM group (1.55 (IQR 1.24-1.90) vs. 1.38 (IQR 1.23-1.78) mg/L, P = 0.009). In addition, no differences were found in medication use and treatment strategies between the two groups. No differences were found in ACS type (P>0.05).

**Table 1:** Study population clinical characteristics in ACS octogenarians with DM and non-DM

**Characteristic, n (%)**	**DM group (n=223)**	**Non-DM group (n=398)**	**P Value**
**General conditions**			
Age (years)	81.74 ± 2.54	81.99 ± 2.21	0.201
Male, n (%)	152 (68.16)	299 (75.13)	0.078
HR (bpm)	75.91 ± 13.74	74.23 ± 14.34	0.156
BMI (kg/m^2^)	24.82 ± 3.30	24.45 ± 3.45	0.193
TG	1.63 ± 0.79	1.54 ± 0.68	0.136
TC	3.86 ± 1.13	3.95 ± 1.11	0.336
LDL-C	2.28 ± 0.64	2.32 ± 0.57	0.423
FBG (mmol/L)	8.52 ± 2.26	6.36 ± 2.56	0.000
HbA1c (%)	8.62 ± 2.17	6.64 ± 1.21	0.000
SBP (mmHg)	138.85 ± 20.74	136.07 ± 22.01	0.124
DBP (mmHg)	69.68 ± 12.04	72.62 ± 12.09	0.004
EF (%)	55.72 ± 8.81	55.35 ± 10.11	0.647
Genesis score	47(IQR 21.5–73)	44 (IQR 18–76)	0.202
GRACE score	138 (IQR 131–153)	138 (IQR 131–151)	0.707
**Risk factors**			
Hypertension	193 (86.55)	286 (71.86)	0.000
Hyperlipoidemia	55 (24.66)	88 (22.11)	0.532
Previous MI	35 (15.70)	81 (20.35)	0.187
Previous stroke	48 (21.52)	78 (19.60)	0.639
CRF	36 (16.14)	39 (9.80)	0.008
Smoking	63 (28.25)	87 (21.86)	0.092
**Renal function indicators**			
CRE (μmol/L)	85.45 (IQR 72.95–112.10)	85.25 (IQR 72.58–99.93)	0.620
BUN (mmol/L)	6.85 (IQR 5.51–9.42)	6.66 (IQR 5.11–8.18)	0.078
Cystatin C(mg/L)	1.55 (IQR 1.24–1.90)	1.38 (IQR 1.23–1.78)	0.009
e-GFR (ml/min/1.73 m^2^)	68.67 (IQR 55.97–82.14)	72.55 (IQR 63.08–81.74)	0.106
**Cardiovascular medications**			
Aspirin	219 (98.21)	384 (96.48)	0.328
Clopidogrel	212 (95.07)	380 (95.48)	0.973
Beta-blocker	149 (66.82)	244 (61.31)	0.201
ACEI/ARB	143 (64.13)	224 (56.28)	0.068
Statin	204 (91.48)	370 (92.96)	0.608
**ACS type**			0.178
STEMI	22 (9.87)	54 (13.57)	
NSTEMI	27 (12.11)	34 (8.54)	
UAP	174 (78.02)	310 (77.89)	
**Treatment strategy**			
PCI (emergency PCI)	146 (13)	268 (39)	0.636
CABG	9	17	0.888
Intensive medcine	58	113	0.524

DM =Diabetes mellitus, HR= Heart rate, BMI= body mass index, TG =triglyceride, TC= total cholesterol, LDL-C= low density lipoprotein-C, FBG=fasting blood glucose, SBP=systolic blood pressure, DBP=diastolic blood pressure, EF=eject fraction, MI=myocardial infarction, CRF=chronic renal failure, CRE=serum creatinine, BUN=urea nitrogen, eGFR=estimated glomerular filtration rate, ACEI=angiotensin converting enzyme inhibitor, ARB=angiotensin receptor blocker, ACS= acute coronary syndrome, STEMI= ST-segment elevated myocardial infarction, NSTEMI= non-ST-segment elevated myocardial infarction, UAP= unstable angina pectoris. PCI=percutaneous coronary angiography, CABG=coronary artery bypass grafting.

### Long- term clinical outcomes

**Figure 1:**
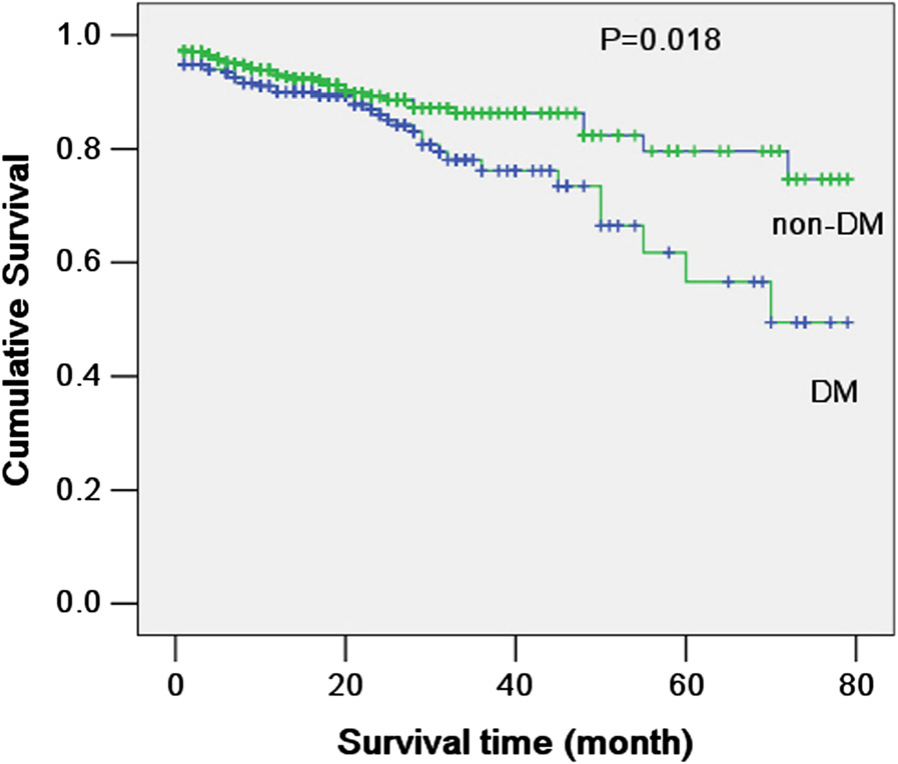
Kaplan-Meier survival rate curves for all-cause death according to diabetes.

The duration of the follow-up was between 13 and 79 months (median 28 months, IQR 16–38 months). During the follow-up period, 40 patients (17.94%) died and 15 patients (6.73%) experienced reinfarction or revascularization, and the rate of MACE was 24.67% in DM group. 44 patients (11.06%) died and 25 patients (6.28%) experienced reinfarction or revascularization, and the rate of MACE was 17.34% in non-DM group. The all cause mortality and MACE rate in DM group were higher than those in non-DM group (P = 0.011, P = 0.037). The rates of reinfarction and revascularization were not significantly different between the two groups (P>0.05). Figure 1 shows the Kaplan-Meier survival curve for all-cause death according to diabetes. There was a significant difference between the two groups (P = 0.018). DM group had a high risk of all-cause death.

**Figure 2:**
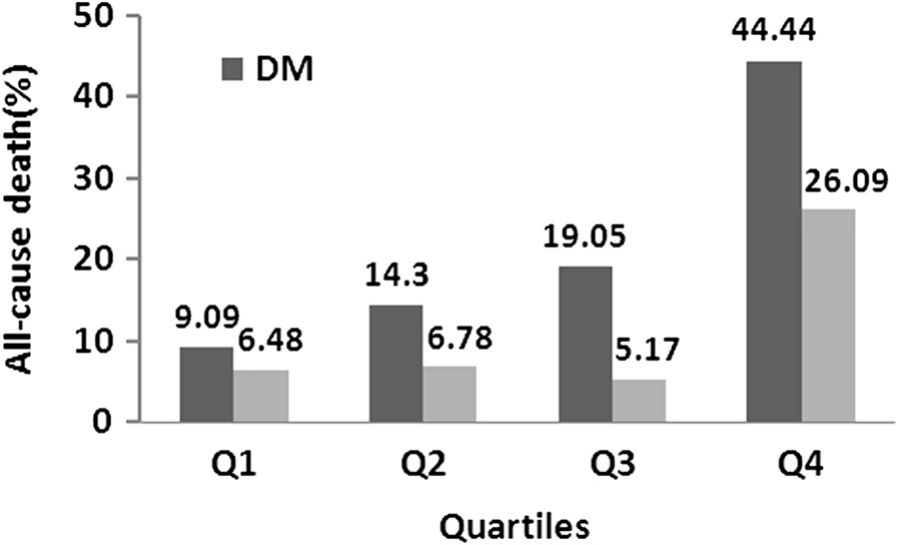
Quartile analysis comparing the concentration of Cys C to the mortality of DM and non-DM group.

Concentration of Cys C relative to all-cause death according to quartile analysis was compared between DM group and non-DM group. Quartiles (Q) ranges of Cys C were: Q1: 1.11(0.52-1.22), Q2: 1.31(1.23-1.43), Q3: 1.59(1.44-1.82), and Q4: 2.23(1.83-5.12) mg/L. As detailed in Figure 2, the mortality of each quarter in DM group was higher than that in non-DM group. The differences increased along with concentration of Cys C, and in ranges Q3 (P = 0.016) and Q4 (P = 0.036) the differences were significant. The mortality increased with the concentration of Cys C and the tendency was significant in DM group (P = 0.0001). While in non-DM group, the mortality was higher than others only in range Q4 (P = 0.01), and the differences in ranges Q1 to Q3 were not significant.

### Risk factors associated with mortality during follow-up

We performed a Cox regression analysis to determine the factors that were associated with all cause death in DM group and non-DM group at the end of the follow-up. After being adjusted for age, gender, heart rate, BMI, TG, TC, LDL-C, FBG, HBA1c, SBP, DBP and EF, the plasma Cys C concentration (OR = 3.32, 95% CI = 1.18-10.92, P = 0.023) was the unique independent predictor for long-term mortality, and the plasma Cys C concentration (OR = 2.47, 95% CI = 1.07-7.86, P = 0.029) and Genesis score (OR = 1.01, 95% CI = 1.00-1.03, P = 0.043) were independent predictors for MACE in DM group.

### Diagnostic power of Cys C and other renal function indicators for mortality during follow-up

**Figure 3:**
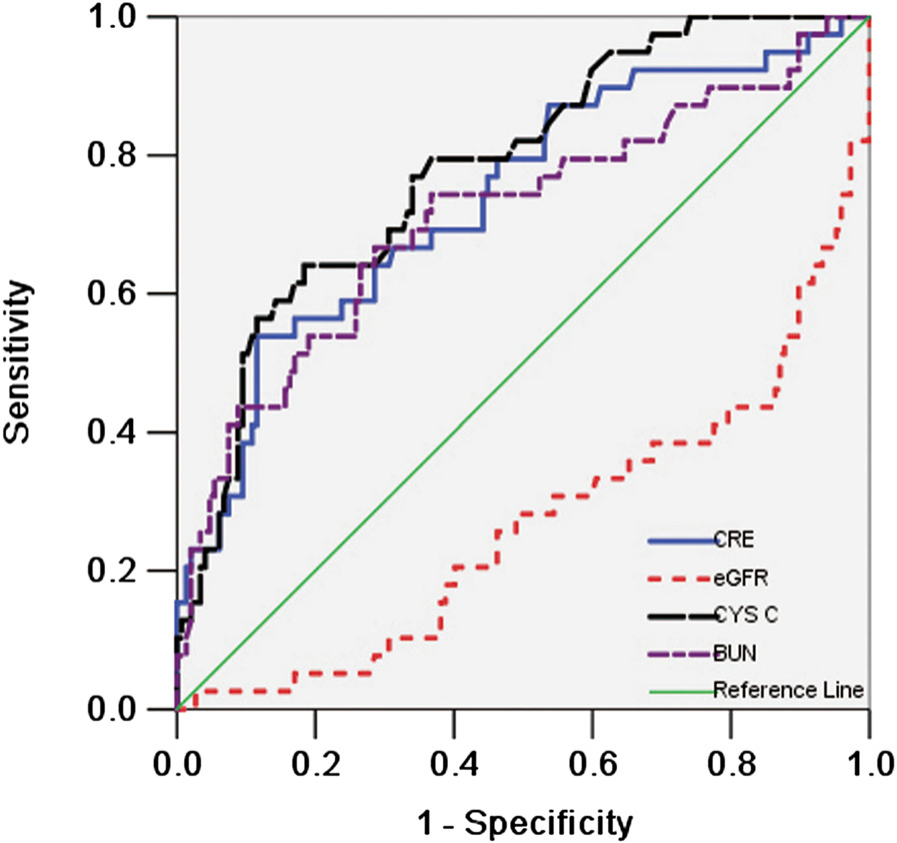
ROC curves for Cys C and other renal function indicators for discrimination between survived and dead patients in DM group.

The respective predictive cut-off values were constructed according to the ROC curves for Cys C and other renal function indicators for discrimination between survived and dead patients in DM group (Figure 3). Using these cut-off points, Cys C showed higher sensitivity and specificity with a greater area under the ROC curve than other biomarkers (Table 2). The predictive cut-off value of Cys C for mortality of DM group was 1.605 mg/L.

**Table 2:** Performances of measures of renal function for prediction of mortality

**Indicators**	**Cut-off point**	**Sensitivity**	**1-specificity**	**95% CI**	**AUC**	**P value**
**Cys C**	1.605	0.718	0.704	0.703; 0.861	0.782	P<0.01
**CRE**	96.9	0.667	0.687	0.645; 0.829	0.737	P<0.01
**BUN**	7.75	0.692	0.66	0.612; 0.813	0.712	P<0.01
**eGFR**	90.2	0.692	0.633	0.172; 0.360	0.266	P<0.01

CI= confidence interval, AUC= areas under the curve. Cys C= Cystatin C, CRE= serum creatinine, BUN= blood urea nitrogen, e-GFR= estimated glomerular filtration rate.

## Discussion

In the present study, we have found that plasma Cys C concentration was an independent predictor for long-term mortality and MACE in ACS octogenarians with DM after controlling for conventional cardiovascular risk factors, but not for those without DM. To the best of our knowledge, our study is the first prospective cohort study to enroll a large number of Chinese ACS octogenarians with DM, the duration of the follow-up is the longest (from 13 month to 79 month) and the follow-up rate is the highest.

Previous studies show that mild renal insufficiency is a major risk factor for CVD and predicts the worst outcomes [18]. In recent years, general studies have indicated that Cys C was proposed as a more reliable marker of renal function than serum creatinine [19], particularly in the detection of small reductions in GFR [20]. Factors that influence serum creatinine levels do not affect Cys C levels. In the present study, we found that among the respective predictive cut-off values, Cys C showed higher sensitivity and specificity with a greater area under the ROC curve than other renal function biomarkers. However, some other studies reported that serum Cys C had limited predictive capacity for the early detection of acute kidney injury after cardiopulmonary bypass surgery in infants and young children. The main reasons lied in the difference in enrolled population. The age in those other studies was younger than 3 year old [21].

Study has demonstrated that high Cys C concentration is related directly to both inflammation and atherosclerosis [22]. Cys C has also been shown to be independent prognostic information in cardiovascular disease in the general population [23-27]. In addition, study has also suggested that patients with higher Cys C concentration appear to have high risk of CAD, and the level of Cys C was also related to long term all cause and cardiovascular mortality in patients referred to coronary angiography [24,28,29]. Consistent studies have reported Cys C concentration was associated with all cause death risk for non-STEMI ACS [30], and prognostic value for patients with STEMI undergoing primary PCI [31]. On the contrary, a study has shown Cys C concentration was not related to the new onset DM [32]. The main reasons lied in the difference in enrolled population and the combined disease. The main diseases in the study were hypertension with pre-diabetes, and the average years were 69.4 year old. In the present study, we found that the prognosis value of Cys C in ACS octogenarians with DM was independent of renal function impaired, and plasma Cys C concentration played the most obvious role and was an effective indicator for all cause mortality. Cys C as a cysteine proteinase inhibitor may play an important role in the pathogenesis of atherosclerosis. Cys C is associated with cardiovascular risk factors as well as inflammation, which may promote atherosclerosis. Some studies have suggested that inflammatory cytokines related to atherosclerosis stimulate lysosomal cathepsins, which may be associated with increased Cys C levels to counterbalance the elastolytic activity of cathepsins [22]. All these effects may cause the worse vascular endothelial damage, inflammation and atherosclerosis in DM patients.

In addition, previous studies show age was a very important risk factor for predicting prognosis of ACS patients in general population. In GRACE study, age was the most important factor affecting in-hospital and six-month discharge mortality, However, for very old patients (age *>*80 years old), the ratio of age score was too high in the GRACE risk score, and all patients enrolled in our study were at very high risk according to GRACE risk score. The need of searching for new biomarkers with better and more accurate profiles to evaluate prognosis of these very high risk ACS patients at the age of > 80 years old has been very intense. Our results indicated, Cys C concentration was an intense biomarker for evaluation of prognosis of Chinese ACS octogenarians with DM and was not influenced by age. This is the first time to define the cutoff value of Cys C concentration for predicting all cause mortality in ACS octogenarians with DM.

As we know, the proportion of ACS with DM in elderly patients is significantly higher than that in the general population, the patients are usually very high-risk groups and have many complications, and the prognosis of them is poor. Therefore the risk stratification in these patients is particularly important. Finding accurate indicators which can be used to determine the prognosis of these patients emerges as a target of interest. In our study, we analyzed the general conditions and comorbidities in DM and non-DM patients, and the ratio of DM in the observed Chinese ACS octogenarians was over one third, which was higher than the prior reports by about 20-30% [33,34]. The Cys C level was significantly different, while severity of coronary artery (Genesis score), GRACE risk score as well as general renal function indicators were not significantly different between DM and non-DM patients. All the enrolled patients had complex, multi-vessel and diffuse coronary artery lesions, and were at very high risk. The results of our study were not completely consistent with the usual concepts that the renal function was obviously poor and coronary artery lesions were complicated in DM patients than those in non-DM patients. It was thus clear that the impact of DM on the renal function and coronary artery lesions severity was small in very old age patients.

Some limitations must be considered. Our study was similar to other single center registries in that it offered observational data on nonrandomized patients. In addition, plasma Cys C levels was measured at a single point in the present study.

## Conclusions

All cause mortality and MACE of ACS octogenarians with DM were higher than those of ACS octogenarians without DM. Plasma Cys C level was an independent predictor for long-term mortality and MACE of ACS octogenarians with DM, while not for non-DM patients. Plasma Cys C level was an accurate biomarker for predicting the long term prognosis for ACS octogenarians with DM.

## Abbreviations

ACEI: Angiotensin converting enzyme inhibitor; ACS: Acute coronary syndrome; ARB: Angiotensin receptor blocker; AUC: Areas under the curve; BMI: Body mass index; BUN: Urea nitrogen; CAD: Coronary artery disease; CAG: Coronary angiography; CABG: Coronary artery bypass grafting; CI: Confidence interval; CRE: Serum creatinine; CRF: Chronic renal failure; CVD: Cardiovascular disease; Cys C: Cystatin C; DBP: Diastolic blood pressure; DM: Diabetes mellitus; EF: Eject fraction; e-GFR: Estimated glomerular filtration rate; FBG: Fasting blood glucose; HR: Heart rate; IQR: Inter quartile range; LDL-C: Low density lipoprotein-C; MACE: Major adverse cardiac events; MI: Myocardial infarction; NSTEMI: Non-ST-segment elevated myocardial infarction; OR: Odds ratio; PCI: Percutaneous coronary angiography; ROC: Receiver operating characteristic curves; SBP: Systolic blood pressure; SD: Standard deviation; STEMI: ST-segment elevated myocardial infarction; TG: Triglyceride; TC: Total cholesterol; UAP: Unstable angina pectoris.

## Competing interests

The authors declare that they have no competing interests.

## Authors’ contributions

ZF, HX, YC designed the research and drafted the manuscript. JG, LC, LG, HL, ZS performed the PCI. WD followed up patients and recorded the results. All authors read and approved the final manuscript.
